# Role of Lipids in the Onset, Progression and Treatment of Periodontal Disease. A Systematic Review of Studies in Humans

**DOI:** 10.3390/ijms17081202

**Published:** 2016-07-25

**Authors:** Alfonso Varela-López, Francesca Giampieri, Pedro Bullón, Maurizio Battino, José L. Quiles

**Affiliations:** 1 Department of Physiology, Institute of Nutrition and Food Technology “Jose Mataix”, University of Granada, Biomedical Research Center, Avda. Conocimiento s/n, 18100 Armilla, Spain; alvarela@ugr.es; 2 Dipartimento di Scienze Cliniche Specialistiche ed Odontostomatologiche (DISCO)-Sez. Biochimica, Facoltà di Medicina, Università Politecnica delle Marche, 60131 Ancona, Italy; f.giampieri@univpm.it (F.G.); m.a.battino@univpm.it (M.B.); 3 Department of Stomalogy, Dental School, University of Sevilla, C/Avicena s/n, 41009 Sevilla, Spain; pbullon@us.es

**Keywords:** diet, fatty acids, polyunsaturated fatty acids (PUFA), nutrition, oral health, periodontitis

## Abstract

The risk of different oral problems (root caries, tooth mobility, and tooth loss) can be increased by the presence of periodontal disease, which has also been associated with a growing list of systemic diseases. The presence of some bacteria is the primary etiology of this disease; a susceptible host is also necessary for disease initiation. In this respect, the progression of periodontal disease and healing of the periodontal tissues can be modulated by nutritional status. To clarify the role of lipids in the establishment, progression, and/or treatment of this pathology, a systematic review was conducted of English-written literature in PubMed until May 2016, which included research on the relationship of these dietary components with the onset and progression of periodontal disease. According to publication type, randomized-controlled trials, cohort, case-control and cross-sectional studies were included. Among all the analyzed components, those that have any effect on oxidative stress and/or inflammation seem to be the most interesting according to current evidence. On one hand, there is quite a lot of information in favor of a positive role of n-3 fatty acids, due to their antioxidant and immunomodulatory effects. On the other hand, saturated fat-rich diets increase oxidative stress as well the as intensity and duration of inflammatory processes, so they must be avoided.

## 1. Introduction

Periodontal disease is a multifactorial pathology featured by the breakdown of periodontal tissues [1]. There is an increasing proportion (from 30% to 65% over the last decades) of adults retaining their teeth until late in life [2], and, nowadays, periodontal disease is a serious problem in older adults [3]. Additionally, the risk of other oral problems (root caries, tooth mobility, and tooth loss) can be increased by the presence of periodontal disease, which has also been associated with a growing list of chronic systemic diseases and impaired cognition [4,5,6,7,8,9,10,11,12,13,14,15,16].

Traditionally, research on the effect of nutrition on oral disease has focused on the dietary effects on the risk of caries [17]. However, it is less understood how the diet affects the development and progression of periodontal disease. A bidirectional correlation has been reported among nutrition, dietary intake, and oral health [18,19]. On one hand, some studies have indicated that oral health status may have implications for dietary intake [20,21,22]. On the other hand, it is largely assumed that the progression of periodontal disease and healing of the periodontal tissues can be modulated by nutritional status. Actually, in spite of the fact that the presence of bacteria is the primary etiology of this disease, a susceptible host is also necessary for disease initiation [23]. Furthermore, many inflammatory conditions and/or diseases, such as type 2 diabetes mellitus (DM), cardiovascular diseases, and rheumatoid arthritis have been related to diet, all of which have been associated with periodontitis [24]. Likewise, nutritional status could affect the integrity of both hard and soft tissues in the oral cavity [23]. First, during teeth development, foods provide a nutritional or systemic effect that may affect the maturation of dentine and enamel. Then, when the teeth have erupted, foods can influence the maintenance of their structure through dietary and topical effects [25]. Regarding all of these implications, it has been hypothesized that “periodontal treatment could be enhanced with the addition of certain nutrients to periodontal therapy, providing a safe method to potentiate the clinical response following treatment” [26]. In particular, it has been observed that the lipid composition of the cell membranes and blood lipoprotein content can be modified through the diet [27,28,29], which has been linked to susceptibility to oxidative damage [30]. Likewise, the response against certain bacterial products can also be modulated by membrane lipid profile [31]. For these reasons, some modifications of dietary patterns affecting lipid profiles could be interesting to both preventing periodontal diseases and improving periodontal health, since all components affected by them are considered key aspects in the pathogenesis of periodontal disease [11].

There are some reviews which provide information about the association between periodontitis and diet components [3,24,25,32,33,34,35,36,37,38,39] although many of them were restricted to certain population groups [3], study types [3,24,34], and/or did not include lipids [3,24,26,35]. However, few attempts of systematic reviews have been carried out [1,3,24,34]. Overall, the systematic approach with the focused question is precluded by a paucity of nutritional interventions and a wide heterogeneity of designs. This paper systemically reviews the literature available on databases until May 2016 on the relationship of lipids with the development and progression of periodontal disease in humans, attending with special interest to dietary interventions and implications of each one for mechanisms involved in theses pathologies.

## 2. Results

**Figure 1:**
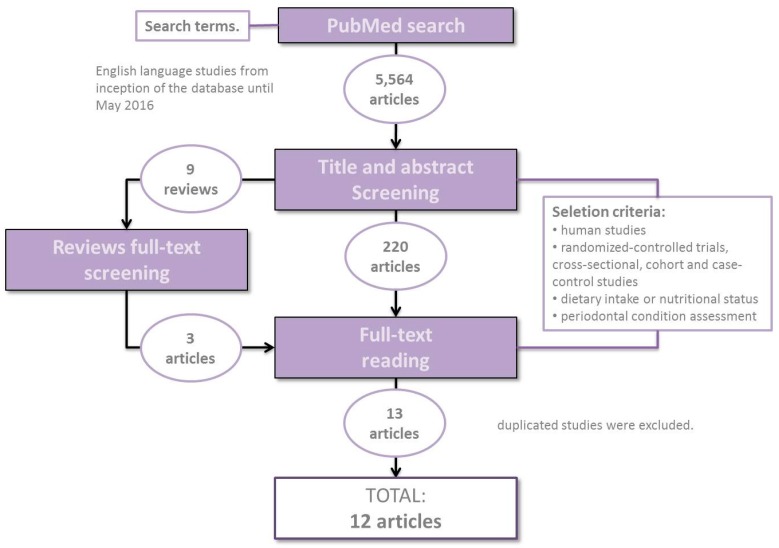
Screening protocol.

A total of 5564 publications were detected in the initial search. After applying inclusion and exclusion criteria, title and abstract screening left 220 available articles. After full-text reading, 13 studies were selected, with one that was duplicated and eliminated (Figure 1).

The relationship between dietary lipids and periodontal disease has been addressed by many reports. These included both observational (Table 1) and experimental (Table 2) studies. Observational studies were represented by two cross-sectional [40,41], a case-control [42], and three cohort studies [43,44,45]. Among the selected cross-sectional studies, dietary fat intake was estimated in two studies [40,41]. In one of them, total dietary intake of carbohydrates, along with many other nutrients, was assessed by a food frequency questionnaire (FFQ) in adults from Japan using associated data from the 2005 National Health and Nutrition Examination Survey (NHANES). After excluding pregnant women and subjects younger than 20 years, the remaining individuals (*n* = 3043) were divided into two groups according to their individual maximum Community Periodontal Index (CPI) values (0–2 or 3–4) to subsequent analyses. Only total dietary fat intake, in terms of total amount and percentage of energy, was available, reporting that fat intake was lower in the group with higher Community Periodontal Index (CPI) (50.8 ± 21 vs. 55.3 ± 23.1 g and 23.2% ± 7% vs. 25.2% ± 7.3% Kcal, *p* < 0.001). After adjusting for confounders in the multivariate logistic regression analysis, only fat as percentage of calories was inversely associated with advanced periodontal disease (adjusted *odds* ratio (OR) = 0.984, *p* = 0.026) [40]. The other available study was performed using a subset of 9182 adult participants in the U.S. NHANES. This study showed a negative association of dietary intake of representative polyunsaturated fatty acids (PUFA) belonging to n-3 series with moderate to severe periodontitis. The adjusted ORs of periodontitis associated with the lowest tertile of n-3 PUFA intake compared with the highest were 0.78 (95% confidence interval (CI): 0.61–1.00, *p* = 0.009) for docosahexaenoic acid (DHA), 0.85 (95% CI: 0.67–1.08, *p* = 0.10) for eicosapetanoic acid (EPA), and 0.86 (95% CI: 0.60–1.23, *p* = 0.28) for γ-linoleic acid (GLA). These associations were changed in a low-way by multivariate adjustment, as well as after DHA, EPA, or GLA dietary supplements users’ exclusion [41].

Results from the single case-control study also had findings supportive of the previously mentioned study. In this study, serum levels of PUFA were compared between people with normal bone height or with a bone loss of more than 3 mm, as determined in a radiographic film. Results showed that patients with bone loss presented higher n-6 PUFA levels than the control group (8.04% ± 0.35% vs. 7.24% ± 0.51% mole; *p* = 0.03), whereas the opposite was observed for n-3 PUFA (2.04% ± 0.17% vs. 2.54% ± 0.41% mole; *p* = 0.01) [42].

All the cohort studies reviewed were based on a subset from the Niigata City (Japan) study. EPA and DHA intakes effect on periodontal disease events over 5 years were evaluated in one of these studies, but only DHA led to relevant results. Individuals who consumed the lowest intake (1st tertile) of DHA presented an incidence rate ratio (IRR) of 1.49 (95% CI: 1.01–2.21) with respect to the highest tertile after simultaneous adjustment for possible confounders [43]. In another study, dietary n-6 to n-3 PUFA ratio was considered as the main predictor to estimate its influence on periodontal disease events after 3 years of follow-up. A high dietary n-6 to n-3 PUFA ratio was significantly associated with a greater number of periodontal disease events in Poisson regression analysis [44]. In the last cohort study, saturated fatty acids (SFA) intake was evaluated with respect to the same outcome. Among non-smokers, it was found that high dietary intake of SFA was associated with more periodontal disease events. In more detail, the multivariate adjusted relative risk (RR) values were 1.00, 1.19 (95% CI: 0.72–1.97), 1.55 (95% CI: 0.95–2.52), and 1.92 (95% CI: 1.19–3.11), in the 1st, 2nd, 3rd, and 4th quartiles of SFA intake, respectively [45].

Among the collected studies, six dietary interventions were found [46,47,48,49,50]. The earliest study was conducted by Campan, et al. [46] on subjects without oral hygiene as a model of gingivitis, where subjects received supplements of fish oil (n-3 PUFA-rich) or olive oil (as placebo). After a short-period (8 days), Gingival Index (GI) was reduced by fish oil (*p* < 0.05), but there were no differences between experimental and control groups. Subsequently, other investigations have been aimed at treating patients with periodontitis. The first one [47], performed on adults with periodontitis, used refined olive oil mixed with corn oil as placebo, and fish oil or borage oil as a source of n-3 (EPA), or n-6 (GLA) PUFA, respectively. Different periodontal parameters were measured at baseline and after treatment (12 weeks). The Modified Gingival Index (MGI), Plaque Index (PI), periodontal probing depth (PPD), and β-glucuronidase levels in gingivocrevicular fluid (GCF). After treatment, subjects treated with borage oil displayed improvement of gingival inflammation (1.04 vs. 0.68, *p* = 0.016). Subjects taking either fish or borage oil alone displayed an improvement in PPD. Statistically significant differences were found only when borage oil and placebo were compared (−0.50 vs. 0.02, *p* = 0.044). No change was observed in GCF or β-glucuronidase levels. Four other interventions were done after a scaling and root planning (SRP) treatment [48,49,50,51]. In one of these studies [48], subjects with moderate and severe chronic periodontitis taking n-3 PUFA supplements (comprising a combination of EPA and DHA) were compared with others taking placebo. After 12 weeks of treatment, several periodontal disease outcomes improved, which included PPD (2.15 ± 0.53 vs. 2.77 ± 0.47 mm, *p* < 0.05), clinical attachment loss (CAL) (2.73% ± 0.98% vs. 3.72% ± 0.62%, *p* < 0.05), GI (2.23 ± 0.57 vs. 1.76 ± 0.63, *p* < 0.05); and sulcus bleeding index (SBI, 1.41 ± 0.30 vs. 1.80 ± 0.39, *p* < 0.05). In addition, most of them improved after 6 weeks. These were PPD (2.61 ± 0.52 vs. 3.19 ± 0.48 mm, *p* < 0.05), SBI (1.65 ± 0.28 vs. 2.06 ± 0.40, *p* < 0.05), and GI (1.32 ± 0.21 vs. 1.58 ± 0.33, *p* < 0.05). However, PI remained unchanged among groups. Another study was carried out for a longer period of time (12 months) on generalized chronic periodontitis patients. In this study, supplements of DHA and EPA had no effect on the percentage of sites with bleeding on probing (BOP), visible PI, PPD, or clinical attachment loss. However, sample size in this research (*n* = 15) was quite reduced [52]. In the next study, the combination of n-3 PUFA and aspirin was tested on advanced chronic periodontitis patients. After 3 and 6 months of treatment, a PPD reduction (−2 mm vs. −1.4 mm at 6 months, *p* < 0.05) and a gain of attachment in n-3 PUFA fed subjects with respect to both baseline and control group were confirmed by statistical analyses (75.3% vs. 28.6% sites with 1 to 3 mm gain, 0.8% vs. 0.1% sites with more of 4 mm gain at 6 months, *p* < 0.05). At 3 and 6 months, the n-3 group showed a significant reduction in salivary receptor activator of nuclear factor κB ligand (RANKL) and matrix metalloproteinase-8 (MMP-8) levels compared with baseline. Levels were also lower than the control group at 6 months (*p* < 0.01). Consumption of supplements containing n-3 PUFA and aspirin led to a significant variation in the frequency of pockets with PPD less than 4 mm (34.3% gain at 3 months and 39.1% at 6 months vs. −26.9% at 3 months and −31.5% at 6 months, *p* < 0.05) [53]. In the remaining study, periodontitis patients also received an aspirin treatment, but in this case, the treatment group was supplemented with DHA while the control group was treated with soy and corn oil supplements. Despite possible aspirin effect, DHA supplements decreased the mean PPD (−0.29 ± 0.13 mm, *p* = 0.03) and GI (−0.26 ± 0.13, *p* = 0.04), although there were no differences in PI or BOP. Along with periodontal health assessment, levels of different molecules related to inflammation were also measured in both GCF and plasma. Results analysis showed lower levels of GCF, high sensitivity C-reactive protein (hsCRP) (−5.3 ± 2.4 ng/mL, *p* = 0.03), and IL-1β (−20.1 ± 8.2 pg/mL, *p* = 0.02), but not IL-6 or systemic hsCRP. Moreover, in this study, the adherence to dietary modification was confirmed by measurement of DHA level in red blood cells (this fatty acid increased from 3.6% ± 0.9% to 6.2% ± 1.6% as a consequence of supplementation).

## 3. Discussion

In spite of the information collected in this review, in many cases, there was little evidence supporting some degree of relationship between periodontal disease and lipid intake. This may be because inferences often come from observational studies, mostly cross-sectionals. Furthermore, many of the reviewed studies have been performed in certain age groups, or with particular diseases or physiological situations. So, translating these findings to the overall population is hampered. In addition, few articles investigated the mechanisms underlying the observed relationships. Moreover, although indices used to evaluate periodontal health are few and mostly accepted (such as Russell’s periodontal index, CPI, or GI), there is a wide variety of periodontitis definitions based on alveolar bone loss (ABL), PPD, CAL, or even bleeding (BOP). Consequently, comparisons among findings from different publications are not easy. Nutritional assessment in observational studies may also be inconvenient. When dietary intakes are estimated by 24-h [40,41] or 3-day recalls [42,43], consequent bias may exist, namely in investigations with small sample sizes. Alternatively, conclusions in many cases are derived only from macronutrient assessment in blood [42], but these values are not always directly related to dietary intake. Both methodological features could help to explain why some papers reported no associations or associations only related to certain age groups. In addition, observed associations could be (at least in part) a consequence of an excess in energy intake or to any nutritional deficiency that might occur when dietary intake is modified far from healthy dietary recommendations. On one hand, experimental studies on macronutrient effects often have a difficult interpretation, particularly those focused on the role of quantitative changes in diet. Namely, increases in lipid content usually lead to hypercaloric diets.

**Figure 2:**
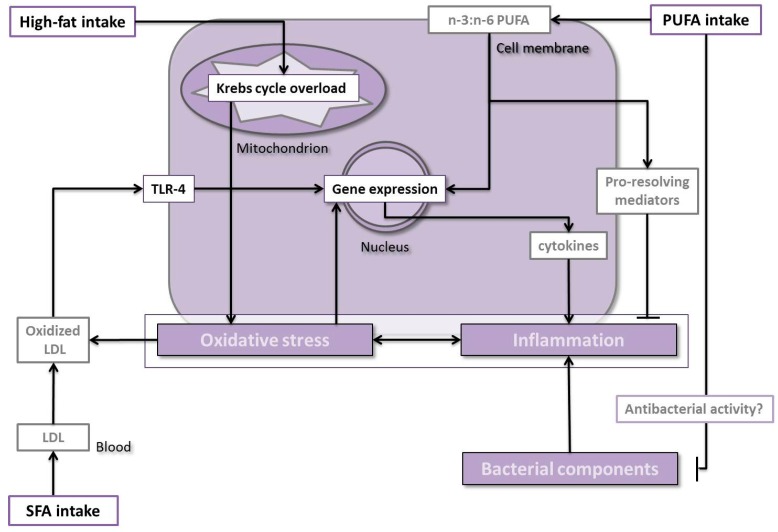
Mechanisms for fat intake effects. Abbreviations: LDL: low-density lipoprotein, PUFA: polyunsaturated fatty acids, SFA: saturated fatty acids, TLR-4: toll-like receptor-4.

Despite these difficulties, it is possible to draw some conclusions. Studies evaluating the possible effects of total amount of fat were only represented by a single study in Japanese adults [40]. It has been proposed that a high fat intake might lead to enhanced oxidative stress because of an overloading of the Krebs cycle, among other reasons [38,39]. Oxidative stress has been associated with periodontal disease exerting its effects by means of direct damage to cells. It has been related through the activation of redox-sensitive transcription factors, leading to the production of pro-inflammatory molecules (Figure 2) which enhance and propagate the inflammatory response, elevating the local levels of oxidative stress [38,39]. Unexpectedly, results from the study mentioned suggested a positive association between periodontal health and fat intake [40]. Several questions could help to explain this rare result. On one hand, a higher percentage of fat in the diet does not necessarily imply a higher intake of calories that can overload the Krebs cycle. On the other hand, dietary fat quality is very important. However, both aspects were not collected by the Japanese study. In this sense, potential effects of different fatty acid types (derived directly from the diet or as supplements) on periodontal disease have been investigated. PUFAs, particularly n-3 PUFAs, have received the most attention, although other fatty acid types have been also studied. Overall, studies conferred a positive role to n-3 PUFA over periodontal health [41,42,43,44], and negative roles to SFA [45]. In turn, results for n-6 PUFA are discussed because it is well known that some of these fatty acids have harmful associations [42,53]. When n-6 PUFAs have been studied directly in comparison with n-3 PUFAs, the former have always reported clearly negative effects [44]. Among studies in favor of these associations, there are some cohort studies, all of them based on subsets from the Niigata City (Japan) study [43,44]. In addition, some nutritional interventions on n-3 PUFA have demonstrated a beneficial effect on periodontitis [48,50]. This was expected because of anti-inflammatory properties have been widely attributed to n-3 PUFA, which are in fact able to down-regulate pro-inflammatory gene expression [53] and the production of pro-resolving lipid mediators, resolvins, maresins, and protectins [33]. Interestingly, it has also been reported that the n-3 PUFAs EPA and DHA have a broad range of antibacterial activities, including the inhibition of putative periodontal pathogens, including *Porphyromonas* *gingivalis*, *Fusobacterium nucleatum*, and *Prevotella intermedia*, among others [54]. The negative association between fat-rich diets and periodontitis found in the Japanese sample could be explained by the role of these fatty acids, since the population of Japan shows a much higher proportion of dietary n-3 PUFA [55] than is present in an average Western diet [56]. Concerning n-6 PUFAs, it is well known that their derived pro-inflammatory prostanoids directly compete with the anti-inflammatory n-3 PUFAs. This fact makes it easy to understand the importance of carefully considering the n-6-to-n-3 PUFA ratio in the diet. In relation to saturated fat, the concern is that an excess metabolically leads to an increased production of low density lipoprotein (LDL) cholesterol. This, when oxidized, forms oxidized LDL, which binds to toll-like receptor 4 (TLR-4) on neutrophils, again activating downstream pro-inflammatory cascades [38], among other things.

It would be possible to conclude that macronutrients that have any effect on antioxidant status or inflammatory processes should be considered for the prevention or improvement of periodontal disease. In this sense, there is quite a body of evidence to consider regarding the positive role of including a high proportion of n-3 PUFA in the diet. On the other hand, those lipids or diets that increase oxidative stress or affect the immune system should be avoided to prevent periodontal disease or to achieve better results after periodontal therapies. These would include saturated fat-rich or hypercaloric diets. However, after analyzing all collected data, there is a need for more studies to confirm the effects of diet, mainly cohort studies with large sample sizes. Additionally, more consensus in periodontitis diagnosis and improvement of dietary intake estimations are needed to enhance our overall understanding. Indeed, problems in the last issue also focus on the importance of carrying out more sensitive experimental investigations and/or methodological approaches. In the future, the design of new clinical trials that combine dietary interventions as periodontal therapies might be the most useful tools to emphasize the importance of diet in these pathologies. Regardless, it would be appropriate to maintain an optimal dietary n-6:n-3 PUFA ratio. This is consistent with the recommendations of the 2011 European workshop on Periodontology that suggested that “the dental team should consider including advice to all patients on increasing levels of fish oils, fibre, fruit, and vegetables, and to reduce levels of refined sugars as part of a periodontal prevention/treatment regime and a general health benefit message” [39,57].

## 4. Material and Methods

### 4.1. Selection Criteria

Inclusion and exclusion criteria for the selection of studies to review were established prior to the start with the literature search. All relevant studies investigating the association between periodontal disease and dietary lipids in humans were included, even when they included only people belonging to certain age groups or subject to any special physiological condition or illness (pregnancy, menopause, DM, and others). For this reason, studies had to assess any periodontal health condition and dietary intake or clear nutritional status markers for the mentioned dietary components. Regarding study design, cross-sectional, cohort, and case-control studies were selected within those belonging to observational type. On the other hand, only randomized-controlled trials (RCTs) were accepted from experimental studies collected.

### 4.2. Information Source and Search Terms

The electronic database of the National Library of Medicine, Washington, D.C. (MEDLINE: PubMed) was used to search potential publications for this review. Firstly, two themes were created and combined by using the Boolean operator “AND”. In turn, the operator “OR” was used to create each theme, combining search terms appearing as either explorer text words or Medical Subjects Headings (Mesh), when they existed and it was not contained within other also used. Asterisk (*) also was used after some terms to search for all terms that begin with the word. The selected periodontal disease related terms were: periodontal, periodontitis, gingivitis and it was also included the following outcome measurement related to periodontal disease: “alveolar bone loss” (Mesh) OR “periodontal diseases” (Mesh) OR “periodontal attachment loss” (Mesh) OR “periodontal index” (Mesh) OR “gingival hemorrhage” (Mesh) OR “periodontal disease” OR periodontitis OR “alveolar bone loss” OR “alveolar bone resorption” OR “tooth attachment” OR “tooth mobility” OR “gingivitis” OR “clinical attachment loss” OR “periodontal attachment level” OR “attachment loss” OR “periodontal pocket” OR “pocket depth” OR “probing depth” OR “bleeding on probing” OR “gingival bleeding” OR “gingival hemorrhage” OR “gingival index” OR “bleeding index” OR “periodontal index”. In addition to this, a second theme related to nutrition or diet was created. Search terms were the next: “food” (Mesh) OR “diet” (Mesh) OR “eating” (Mesh) OR “nutrition surveys” (Mesh) OR “nutrition assessment” (Mesh) OR “nutrition therapy” (Mesh) OR “nutrition processes” (Mesh) OR “nutritional status” (Mesh) OR nutrition* OR nutrition OR nutrient* OR nutrient OR food OR dietary OR diet* OR intake OR intakes OR consumption* OR consumption OR ingestion OR eating.

### 4.3. Search Strategy

The search strategy was focused on English-written studies from inception of the database until May 2016. A comprehensive literature search was run independently by two of the review authors. Firstly, a screening adjusted for higher sensitivity (i.e., without restrictive search items) of title and abstract was carried out. Reviews that could mention studies related to the established selection criteria were collected, and a screening was done to find new additional articles that would follow the rest of the process; Secondly, full-texts were obtained and after full-text screening; those articles that did not accomplish the proposed selection criteria were excluded. Duplicated studies were removed; Moreover, full-text was screened in the web for articles whose title suggested that they were related to these review objectives; Finally, when there were disagreements or inconsistencies relative to the inclusion of some publications or data, all authors discussed the issue to eventually achieve mutual consensus.

### 4.4. Data Collection Process, Data Items, and Summary Measures

Specific data about populations, interventions, study designs, as well as outcomes relevant for this review’s questions were extracted from articles. Data from eligible studies were independently evaluated. When they were available, mean, mean differences and standard deviations of outcome measurements (like clinical outcomes for periodontal disease, nutritional status, or dietary intakes), were included in our description of the results. In the same sense, association measurements were also collected, such as correlation or regression coefficients, IRRs, ORs, RRs and their corresponding 95% CI, as well as significance levels considered or *p*-values, but only if significant associations and/or differences were found.

### 4.5. Quality Assessment and Risk of Bias

Subsequently, potentially-relevant publications were read in full to determine their quality, mainly through the risk of bias assessment. Several criteria depending on the study design were applied to determine methodological quality of each publication. In the case of observational studies, evaluation and strategies to deal with them were considered in addition to risk of bias. According to the prevention of confounding effects, the employment of randomization, only in clinical trials, matching, or restriction, were explored at the design level, while the use of multivariate analysis, stratification, or frequency matching was appraised at the data analysis level. As to the risk of bias, it four types of bias have been proposed: selection, detection, attrition, and reporting bias [58]. Other authors have simplified this classification into two categories: selection and information bias [59]. Regardless, critical issues for bias prevention are different between design types. In cross-sectional studies, three points were mainly evaluated: sample randomization; reliability and objectivity of variables assessment; as well as definition of group if comparisons were made. In case–control studies, objectivity and reliability of evaluations of subjects was also taken into account. For this study type, category definitions and exposures evaluation method also were included. Lastly, in cohort studies, exposure and status definition, measurement, differences in duration, reliability, and objectivity of outcome assessment were considered, as well as the presence of differences in intensity of medical surveillance, loss of follow-up, and missing data treatment. In addition, possible differences in outcomes or exposure to risk factors between subjects who drop out and those who stay in the study were taken into account. Inclusion criteria and/or sources of data or individuals’ background were considered for the establishment of sample external validity and population set represented. Additionally, grouping criteria in data analysis were taken into account, when they were presented, especially if they were selected post hoc from alternative options.

On the other hand, intervention trials were assessed according to Cochrane guidelines, which take into account five type of bias: selection, performance, detection, attrition, and reporting bias [60]. Based in its recommendations, the following domains were evaluated: sequence generation (randomization), allocation concealment, blinding of participants, operators and/or examiners, incomplete outcome data, and selective reporting. With regard to the results of this assessment, degree of risk (high, low, or unclear) was established for each type of bias, as well as its possible magnitude and direction. The risk of bias and its possible effects were summarized for each outcome within each study. Finally, it was summarized for the review as a whole, where it was possible.

## Figures and Tables

**Table 1:** Observational studies on lipids association with periodontal disease.

Reference; Study Type	Subjects; Age	Main Outcomes/Groups Compared	Exposures	Main Results/Conclusion
Hamasaki, et al., 2016 [40]; CS	3043 NHANES participants (Japan) ≥20 years	Adjusted OR of CPI = 3–4	Dietary intake of total fat (wt and %E)	Negative association with dietary intake of fat in %E
Naqvi, et al., 2010 [41]; CS	9182 NHANES 1999–2004 participants (USA); ≥20 years	Adjusted OR of periodontitis ^1^	Dietary intake of FA	Negative association with n-3 PUFA, DHA, EPA and GLA
Requirand, et al., 2000 [42]; CC	105 patients (France) 41.1 ± 2.6 years/43.4 ± 6.6 years	Suffered from bone loss ≤3 mm on several teeth vs. normal bone height-periodontium	Serum levels of PUFA	n-6 PUFA were higher in patients with bone loss, whereas n-3 PUFA were lower
Iwasaki, et al., 2010 [43]; C	55 Niigata study participants (Japan); 74 years	IRR of periodontal disease events ^2^	Dietary intakes of DHA, and EPA	Negative association with DHA intake
Iwasaki, et al., 2011 [44]; C	235 Niigata study participants (Japan); 75 years	Adjusted RR of periodontal disease events ^3^	Dietary intakes energy- adjusted of n-6 and n-3 PUFA and n-6/n-3 PUFA ratio	Positive association with n-6/n-3 PUFA ratio
Iwasaki, et al., 2011 [45]; C	264 Niigata study participants (Japan); 75 years	Adjusted RR of periodontal disease events ^3^	Energy-adjusted dietary intakes of SFA	Positive association

^1^ PPD ≥ 4 mm and AL ≥ 3 mm in any mid-facial or mesial tooth; ^2^ CAL ≥ 6 mm in ≥2 teeth and PPD ≥ 5 mm in ≥1 site; ^3^ number of teeth with AL ≥ 3 mm/year. *Abbreviations*: %E: percentage of Energy; AL: attachment loss; C: cohort study; CC: case-control; CPI: Community Periodontal Index; CS: cross-sectional study; DHA: docosahexaenoic acid; EPA: eicosapentanoic acid; FA: fatty acid; GLA: γ-linoleic acid; IRR: incidence rate ration; NHANES: National Health and Nutrition Survey; OR: Odds ratio; PUFA: polyunsaturated fatty acids; RR: relative risk; SFA: saturated fatty acids; wt: weight; y: years; PPD: periodontal probing depth; CAL: clinical attachment loss.

**Table 2:** Experimental studies on lipids effect on periodontal disease.

Reference; Study Type	Subjects; Age	Experimental Treatments (Duration)	Analytic Measurement	Main Results/Conclusions
Campan, et al., 1997 [46]; RCT	37 healthy volunteers with intensive oral hygiene for 14 days	Oral hygiene abstention (29 days), in combination with supplementation with fish oil or olive oil as placebo (last 8 days)	PI, GI, PBI, and gingival levels (only in 10 volunteers) of AA, EPA, DHA, DPA, PGE2 and LTB4	Fish oil supplements reduced GI, but there are no differences between experimental and control group. LTB4 was lower fish oil treated subjects
Rosenstein, et al., 2003 [47]; RCT (DB)	30 subjects with periodontitis; 18–60 years	Supplementation with EPA or borage oil, both, or a mixture of olive and corn oil as placebo (12 weeks)	PI, MGI, BOP, PPD and CAL and salivary RANKL and MMP-8	Supplementation with borage oil or EPA improved PPD, but only borage oil effect was significant respect to placebo. Additionally, it also improved MGI
Deore, et al., 2014 [48]; RCT (DB)	60 subjects with moderate and severe chronic periodontitis; 45.4 ± 4.9/44.5 ± 5.2 years	Supplementation with n-3 PUFA or placebo; after SRP (6 or 12 weeks)	ABL, *P. gingivalis* identification, serum FA profile	Treatment reduced PPD and salivary RANKL and MMP-8 levels; and increased CAL
Martinez, et al., 2014 [49]; RCT (DB)	15 patients with generalized chronic periodontitis (43.1 ± 6/46.1 ± 11.6 years)	Supplementation with n-3 PUFA or placebo; after SRP (12 months)	% BOP, visible plaque index, PPD and CAL	No effect
El-Sharkawy, et al., 2010 [50]; RCT (DB)	80 subjects with advanced chronic periodontitis; 30–70 years	Supplementation with fish oil and aspirin or placebo; after SRP (3 or 6 months)	PI, GI, OHIS, BOP, SBI, PPD, CAL and serum levels of CRP	Supplementation with borage oil or EPA improved PPD, but only borage oil effect was significant respect to placebo. Additionally, it also improved MGI
Naqvi, et al., 2014 [51]; RCT (DB)	46 subjects with moderate periodontitis; adults	Supplementation with DHA or soy/corn oil capsules, in combination with aspirin (3 months)	GI, PI, BOP, PPD, GCF levels of hsCRP, IL-6 and IL-1β, systemic inflammatory markers plasma levels, and erythrocytes fatty acids	Supplementation with DHA decreased mean PPD and GI. This was accompanied by lower hsCRP and IL-1β levels in GCF

*Abbreviations:* AA: arachidonic acid; ABL: alveolar bone loss; BOP: bleeding on probing; CAL: clinical attachment loss; CRP: C-reactive protein; d: days; DB: double-blind; DHA: docosahexaenoic acid; EPA: eicosapentanoic acid; FA: fatty acid; GCF: gingivocrevicular fluid; GI: gingival index; hsCRP: high sensitive C-reactive protein; IL-6: interleukin-6; IL-1β: interleukin-1β; LTB4: leukotriene B4; m: months; MGI: modified gingival index MMP-8: matrix metalloproteinase-8; OHIS: oral health index; PBI: papillary bleeding index; PGE2: prostaglandin E2; *P. gingivalis: Porphyromonas gingivalis;* PI: plaque index; PPD: periodontal probing depth, PUFA: polyunsaturated fatty acids; RANKL: receptor activator of nuclear factor κB ligand ; RCT: randomized-controlled trial; SBI: sulcus and bleeding index SRP: scaling and root planning; y: years; w: weeks.

## Abbreviations

BOP	bleeding on probing
CAL	clinical attachment loss
CI	confidence interval
CPI	Community Periodontal Index
DHA	docosahexaenoic acid
DM	diabetes mellitus
EPA	eicosapentanoic acid
FFQ	food frequency questionnaire
GCF	gingivocrevicular fluid
GI	Gingival Index
GLA	γ-linoleic acid
hsCRP	high sensitivity C-reactive protein
IRR	incidence rate ratio
LDL	low density lipoprotein
Mesh	Medical Subjects Headings
MGI	Modified Gingival Index
MMP-8	matrix metalloproteinase-8
NHANES	National Health and Nutrition Survey
OR	odds ratio
PI	Plaque Index
PPD	Periodontal probing depth
PUFA	polyunsaturated fatty acid
RANKL	receptor activator of nuclear factor *κ*B ligand
RCT	randomized-controlled trial
RR	relative risk
SBI	Sulcus Bleeding Index
SFA	saturated fatty acids
SRP	scaling and root planning
TLR-4	toll-like receptor 4
